# Editing the genome of *Aphanomyces invadans* using CRISPR/Cas9

**DOI:** 10.1186/s13071-018-3134-8

**Published:** 2018-10-23

**Authors:** Muhammad Majeed, Hatem Soliman, Gokhlesh Kumar, Mansour El-Matbouli, Mona Saleh

**Affiliations:** Clinical Division of Fish Medicine, University of Veterinary Medicine, Vienna, Austria

**Keywords:** CRISPR/Cas9, Dwarf gourami, Epizootic ulcerative syndrome, Oomycetes, Point mutation, Proteases, Virulence

## Abstract

**Background:**

The clustered regularly interspaced short palindromic repeats (CRISPR)/CRISPR-associated protein 9 (Cas9) system is increasingly being used for genome editing experiments. It is a system to add, delete and/or replace parts of a gene *in situ* in a time- and cost-efficient manner. The genome of many organisms has been edited using this system. We tested the CRISPR/Cas9 system in *Aphanomyces invadans*, an oomycete, which is the causative agent of epizootic ulcerative syndrome (EUS) in many fish species. Extracellular proteases produced by this oomycete are believed to play a role in EUS virulence.

**Methods:**

We designed three single guide-RNAs (gRNA) to target *A. invadans* serine protease gene. These gRNAs were individually combined with the Cas9 to form ribo-nucleo-protein (RNP) complex. *A. invadans* protoplasts were then transfected with RNP complexes. After the transfection, the target gene was amplified and subjected to sequencing. Zoospores of *A. invadans* were also transfected with the RNP complex. Three groups of dwarf gourami (*Trichogaster lalius*) were then experimentally inoculated with (i) non-treated *A. invadans* zoospores; (ii) RNP-treated *A. invadans* zoospores; and (iii) autoclaved pond water as negative control, to investigate the effect of edited serine protease gene on the virulence of *A. invadans in vivo*.

**Results:**

Fluorescence microscopy showed sub-cellular localization of RNP complex in *A. invadans* protoplasts and zoospores. Sequencing results from the protoplast DNA revealed a point mutation in the target gene. A matching mutation was also detected in zoospores after similar treatment with the same RNP complex. *In vivo* results showed that the CRISPR/Cas9-treated *A. invadans* zoospores did not produce EUS clinical signs in the fish. These results were then confirmed by histopathological staining of the muscle sections using Gomori’s methenamine silver nitrate and hematoxylin and eosin stains.

**Conclusions:**

Results obtained in this study indicate that the RNP complex caused effective mutation in the target gene. This hindered the production of serine protease, which ultimately impeded the manifestation of EUS in the fish. Our methods thus establish a promising approach for functional genomics studies in *A. invadans* and provide novel avenues to develop effective strategies to control this pathogen.

## Background

Oomycetes are a group of parasites that are widespread and have the ability to invade a wide range of hosts [1]. Three oomycete genera, namely *Achlya*, *Aphanomyces* and *Saprolegnia*, have been described as the most devastating pathogens of aquatic organisms [2–4]. *Aphanomyces* alone contains approximately 35–40 species, some of which are specialized pathogens of plants and animals while others are saprotrophic or opportunistic pathogens growing on animals and plant debris [5, 6].

The species *Aphanomyces invadans* has been identified as the primary cause for epizootic ulcerative syndrome (EUS), which is an important seasonal condition implicated in mass-mortalities of cultured and wild fish in many countries [7, 8]. As soon as the free-swimming zoospore, the infective stage of *A. invadans*, finds a fish host, it germinates into vegetative non-septate hyphae, which invade the fish skin and muscular tissue and may also reach the internal organs [9, 10]. Then, the primary zoospores develop in the sporangium in fish tissues and are released at its tip where they form a spore cluster [11]. The primary spores transform into motile, biflagellate secondary zoospores. The life-cycle is completed once they find new fish to invade [12].

In addition to the distinctive mycotic granulomas in internal tissues, infected fish display distinct dermal lesions appearing as red spots, blackish burn-like marks or deeper ulcers with red centers and white rims [7, 13–15]. More than 125 fish species have been reported to be affected by *A. invadans* [16]. Because of its epizootic nature, broad fish host range and potential impact on cultured and wild fisheries, EUS is officially recognized as a reportable disease by the World Organization for Animal Health [17].

Although a large amount of data on the gross and microscopic clinical lesions of EUS and the involvement of different pathogens during the course of the disease already exist [9, 16–18], there still is a paucity of information regarding pathogenicity, host-parasite interactions, molecular mechanisms of the disease and the functional genomics of *A. invadans*. Similar to many pathogenic microorganisms, proteases secreted by *A. invadans* are key in the pathogenesis of EUS [19]. Different extracellular proteins, most of which are proteases, secreted by *A. invadans*, have recently been identified and at least one of these proteases (serine protease belonging to peptidase_S8 domain) is believed to be involved in the degradation of the fish immunoglobulin M [20]. Serine proteases have long been thought of as the virulence factors in many of the true bacteria and fungi [21]. A serine protease secreted by the fish-pathogenic oomycete *S. parasitica* has been reported as able to degrade the fish IgM [22]. Accordingly, these genes were our target for gene editing to investigate their role in *A. invadans* pathogenesis.

Genome editing is a group of technologies that allow the scientists to make precise changes by adding, removing and/or altering genetic material at particular locations in the genomes of eukaryotic cells [23–25]. In addition to the currently used techniques for genome editing, such as zinc finger nucleases (ZFN) and transcription activator-like effector nucleases (TALEN), a relatively recent technique known as clustered regularly interspaced short palindromic repeats (CRISPR)/CRISPR-associated protein (CRISPR/Cas) system is already revolutionizing our ability to interrogate gene functions and can potentially be used clinically to correct or introduce genetic mutations [24, 26–28].

According to the current classification of CRISPR-*cas* loci the CRISPR/Cas system has been grouped into six distinct types (I-VI). Each type employs a unique set of Cas proteins along with CRISPR RNA (crRNA) for CRISPR interference [29]. The Type II CRISPR system employs a single DNA endonuclease, Cas9, to recognize double-stranded DNA (dsDNA) substrates and cleave each strand with a distinct nuclease domain [30–33]. The use of CRISPR/Cas9 as an RNA-programmable DNA targeting and editing platform is simplified by a synthetic single guide RNA (gRNA) mimicking the natural dual trans-activating CRISPR RNA (tracrRNA)-crRNA structure [34].

Because of its simplicity, specificity and versatility, the CRISPR/Cas system has recently emerged as a powerful tool for genome engineering in various species [35–38] including fungi [39–41] and bacteria [42–45]. In oomycetes, CRISPR/Cas9-mediated gene disruption and gene replacement were performed to test the function of specific genes in *Phytophthora sojae*, the causative agent of stem and root rot of soybeans [46–48].

The aim of the present study was to use CRISPR/Cas9 system to edit the genome of *A. invadans* by targeting the serine protease gene *in vitro* and study the effect of this genome editing on the virulence and pathogenicity of the *A. invadans in vivo*.

## Methods

### *Aphanomyces invadans* growth and zoospore production

*Aphanomyces invadans* strain 9701 was used in this study. It is routinely grown on glucose-peptone-yeast (GPY) agar and maintained on glucose-peptone (GP) agar at 25 °C according to Lilley et al. [9]. For zoospore production, agar plugs (4 mm) were aseptically transferred from stock cultures to GPY agar and grown for 4–5 days at 25 °C. A piece of agar containing hyphae from the growing edge of a colony was then aseptically transferred to GP broth supplemented with oxolinic acid (100 μg/ml) and incubated at 25 °C for 3–4 days. The resulting mycelial wads were then aseptically removed from the broth, rinsed four times with sterile distilled water and transferred into Petri dishes containing 20 ml filtered autoclaved pond water (APW) (one part filtered river water combined with one part distilled water) for 24 h. The production of secondary zoospores was microscopically monitored. The produced zoospores were counted using a hemocytometer.

### Design of gRNAs and the formation of ribo-nucleo-protein (RNP) complexes

The Eukaryotic Pathogen CRISPR Guide RNA Design Tool (EuPaGDT) [49] was used to design the gRNAs for serine type protease of *A. invadans* (gene ID: 20088162). Secondary structures were checked using RNAstructure, a web server tool for RNA secondary structure prediction [50], while off-targets were checked using FungiDB as per Fang & Tyler [48]. Sequences of chosen gRNAs were submitted to Integrated DNA Technologies, Skokie, USA, for *in vitro* synthesis of crRNA. tracrRNA labeled with a fluorescent dye ATTO^TM^ 550 and Alt-R^TM^ (*Streptococcus pyogenes*) S.p. Cas9 nuclease 3NLS were purchased from Integrated DNA Technologies. crRNA and tracrRNA were separately resuspended in nuclease-free duplex buffer (Integrated DNA Technologies) to produce 100 μM stock solution. Subsequently, 30 μl from each were mixed and annealed at 95 °C, according to manufacturer’s instructions, and then cooled at room temperature (RT). Each gRNA (1 μM) was combined with Cas9 (1 μM) in Opti-MEM media supplemented with GlutaMax (Thermo Fisher, Vienna, Austria) to form the RNP complexes. After incubation of the mixture for 5 min at RT, the complex was ready for transfection.

### PCR primers

Three PCR primer sets (product sizes 211, 214 and 209 bp) corresponding to each gRNA were designed (Table 1) based on the *A. invadans* target gene (GenBank: XM_008877667) to amplify the target fragment and observe the genomic DNA mutation caused by Cas9 and gRNAs in the target gene *via* sequencing of the amplified products. Possible homology of the selected oligonucleotides was excluded using BLASTn analysis.

**Table 1:** PCR primers designed for each guide RNA. Three PCR primer sets corresponding to each gRNA were designed based on the *A. invadans* target gene (GenBank:  XM_008877667) to detect the genomic DNA mutation, caused by CRISPR/Cas9

	Name	Position	Expected product size (bp)	Sequence (5'- 3')
Primer set 1	AiCr1F	35–54	211	GACTCCGACCTTGACGATGC
	AiCr1R	227–246		AAAGAGGAATGAGGCGGAGG
Primer set 2	AiCr2F	884–904	214	CCCACACCATGGGAACGATTG
	AiCr2R	1080–1098		TTGAGACGAGCCCCACGAG
Primer set 3	AiCr3R	1572–1591	209	GTTCGGCCGTATTAACGCCA
	AiCr3F	1763–1781		CTTGGTACATGGCTGCCCA

### Protoplast isolation

*Aphanomyces invadans* protoplasts were isolated following the method described by Fang et al. [47] with minor modifications. Briefly, *A. invadans* mycelia were peeled off of the GPY plates (or the plug of germinated zoospores was isolated from the broth), rinsed first in water and then in 0.8 M mannitol (Sigma-Aldrich, Vienna, Austria) for 10 min each. Plasmolysis was carried out using 0.8 M mannitol for 20 min. Subsequently, the digestion process was completed using 20 ml enzyme solution [0.4 M mannitol, 20 mM KCl, 20 mM 2-(N-morpholino) ethanesulfonic acid (MES) (pH 5.7), 10 mM CaCl_2_, 0.5% lysing enzymes from *Trichoderma harzianum* and 0.5% CELLULYSIN® Cellulase (Sigma-Aldrich)] for 45 min on a 50× *rpm* shaker. Digestion efficiency for the release of protoplasts in the solution was checked under a light microscope (Olympus BX53, Tokyo, Japan). The digestion product was then filtered through 70 μm Corning® cell strainers (Sigma-Aldrich) to remove mycelial debris. The flow-through was collected and centrifuged at 1200× *g* for 2 min. The supernatant was then discarded and the pellet washed by slowly adding up to 5 ml of W5 solution (5 mM KCl, 125 mM CaCl_2_, 154 mM NaCl and 177 mM glucose). Excess W5 solution was discarded after spinning the tube at 1200× *g* for 2 min. W5 solution (2 ml) was added and protoplasts were placed on ice for 20 min. Protoplasts were spun again at RT and excess W5 solution was removed. Protoplasts were then resuspended by gently adding 2 ml of MMG solution [0.4 M mannitol, 15 mM MgCl_2_ and 4 mM MES (pH 5.7)] at RT for 10 min. The protoplasts were counted and optimized to 10^5^ per ml.

### Transfection of *A. invadans* protoplasts

Polyethylene glycol (PEG)-mediated RNP delivery into the protoplasts was carried out following the protocol described by Malnoy et al. [51] with minor modifications. Briefly, 200 μl of resuspended *A. invadans* protoplasts (20,000 protoplasts) were mixed with 12 μl RNP and incubated at RT for 10 min. Another 200 μl protoplast suspension was mixed with 12 μl opti-MEM medium and incubated for 10 min at RT to be used as a control. An equal volume of PEG 4000 (Sigma-Aldrich) was added, gently mixed and incubated at RT for a further 20 min. W5 solution (400 μl) was then added, mixed and kept at RT for 10 min, followed by the addition of 800 μl W5 solution. After another 10 min of incubation at RT, the protoplasts were centrifuged at 50× *g* for 5 min. The supernatant was discarded and the pellet was washed with 500 μl W5 solution and incubated at RT overnight. To observe the efficient delivery of RNP into the protoplasts, 15 μl of each transformation mixture including control was sampled and examined by fluorescence microscopy. Regeneration media (double strength GPY broth) was then added and the mixture was divided into two parts; one part was used for DNA extraction and the other incubated for 3–4 days at 25 °C to ensure that the RNP did not affect the viability of the protoplasts.

### Transfection of *A. invadans* zoospores

*Aphanomyces invadans* zoospores (4000 zoospores in 400 μl suspension) were transfected by adding 24 μl of RNPs and incubated at RT for 60 min. Non-treated zoospores were used as control. Both treated and non-treated zoospores (200 μl) were then subjected to DNA extraction, PCR and sequencing to observe the mutation. The other 200 μl of zoospores were added to GYP medium and incubated at 25 °C for 4–5 days to demonstrate the effect of RNP complex on the viability and growth of the treated zoospores.

### Detection of targeted mutation

DNA from RNP-treated and non-treated *A. invadans* protoplasts and zoospores were isolated using Qiagen DNeasy Blood & Tissue Kit (Qiagen, Vienna, Austria) according to the manufacturer’s instructions. To exclude the possibility of mutations caused during PCR amplification, Platinum SuperFi Green PCR Master Mix (Invitrogen, Vienna, Austria) was used in all PCR reactions. The amplification reaction mixture consisted of 12.5 μl Platinum SuperFi Green PCR Master Mix, 15 pmol of each sense and antisense primers, 2.5 μl DNA template and PCR grade water to a final volume of 25 μl. The reaction mixture was subjected to 35 amplification cycles comprising: denaturing at 98 °C for 10 s, annealing at 57 °C for 10 s and extension at 72 °C for 30 s. The amplification cycles were preceded by an initial denaturing step of 98 °C for 30 s followed by an extended elongation step of 72 °C for 5 min. Amplified products were analyzed on 1.5% agarose gel.

For sequencing, PCR products were purified using MinElute gel extraction kit (Qiagen) as per the manufacturer’s instructions. The purified products were either directly sequenced or incubated with Taq polymerase buffer, dATP and Taq polymerase enzyme at 72 °C for 20 min to add 3’ adenines to the blunt-end fragments for TA cloning. For TA cloning, PCR products were cloned into the pCR® 4-TOPO® vector using TOPO TA cloning® kit (Invitrogen) according to the manufacturer’s instructions. The purified PCR products and recombinant plasmids were subjected to sequencing in a commercial sequencing laboratory (LGC Genomics, Berlin, Germany). Sequences were subjected to BLASTn analysis for sequence identity and then compared with the *A. invadans* target gene (GenBank: XM_008877667) to detect the mutation.

### *In vivo* investigation of the effect of CRISPR/Cas9-treated *A. invadans* zoospores on dwarf gourami fish

Dwarf gourami (*Trichogaster lalius*) were purchased from a local pet shop in Austria. Five random fish were taken for bacteriological and parasitological analyses to rule out the possibility of other infections prior to the *in vivo* experiment. Furthermore, muscle samples were taken from these 5 fish for PCR to rule out the possibility of prior infection with *A. invadans* according to Vandersea et al. [52]. Fish (*n* = 180, mean total length 4.5 ± 0.5 cm and mean weight 5 ± 0.5 g) were kept in aquaria containing de-chlorinated water and equipped with air stones and aquarium heaters to provide aeration and maintain the temperature at 24 ± 1 °C. Water was changed (25%) weekly to maintain water quality. After 3 weeks of acclimatization, fish were divided into three groups. Each group had three replicates and each replicate contained 20 fish. The fish were anaesthetized using MS-222 (Sigma-Aldrich) 150 ppm prior to zoospore injection. After transfection of *A. invadans*’ zoospores with RNP complex for 60 min, each fish in the RNP-2-treated group was intramuscularly injected with 0.1 ml of 10,000 zoospores/ml spore suspension (1000 zoospores/fish). Similarly, in the positive control group, each fish received 0.1 ml of 10,000 zoospores/ml non-treated *A. invadans* zoospores intramuscularly. The negative control group was inoculated with an equal volume of APW. All fish were monitored daily for characteristic EUS clinical signs for 30 days. Fish with clinical signs were collected as appropriate. Similarly, fish were sampled from both RNP-2-treated and negative control groups. Collected fish were euthanized and subjected to histological and PCR examinations for confirmation of *A. invadans*. Skin and muscle tissues were excised from lesion area and divided into two parts. One part was subjected to DNA extraction and the second part was fixed in 10% phosphate-buffered formalin for histology.

### Histopathology

Tissue samples were processed through an automatic tissue processor and embedded in paraffin. The paraffin blocks were then sectioned at 4 μm thickness and stained with Gomori’s methenamine silvernitrate (GMS) and hematoxylin and eosin (H&E) stains [53]. Stained sections were viewed under a light microscope (Olympus BX53). Photomicrographs were taken with a camera (Olympus DP73) attached to the microscope and processed using CellSens Standard software of Olympus.

## Results

### Design of *A. invadans* specific gRNAs

Targeting a serine type protease of *A. invadans* [20], gRNA candidates were suggested by the gRNA design tool and further filtered for secondary structure and off-targets. Based on their high total and efficiency scores and zero off-target hits, three gRNA candidates were selected (Table 2), synthesized and used for the formation of 3 separate RNP complexes.

**Table 2:** Guide RNAs used in this study. The Eukaryotic Pathogen CRISPR Guide RNA Design Tool was used to design the gRNAs for serine type protease of *A. invadans* (gene ID: 20088162). Based on their high total and efficiency scores and zero off-target hits, three gRNAs were selected for genome modification experiment

gRNA id	gRNA sequence (PAM “NGG”)	Total score	Efficiency score	On-target hits in the genome (perfect-match | non-perfect-but-PAM-match)	Off-target hits (perfect-match | nonperfect-match)
1. Aphanomyces-invadans_106	GCT TAC CAG ATG GAA TGA CG | TGG	0.69	0.65	2 | 0	0 | 0
2. Aphanomyces-invadans_923	GTG TAG CCC CAG AAG CCC AA | TGG	0.56	0.57	1 | 1	0 | 0
3. Aphanomyces-invadans_1730	GAT GGA ACC CAA CAA CTG TG | TGG	0.53	0.65	1 | 0	0 | 0

*Abbreviation*: *PAM* protospacer adjacent motif

### Transfection of protoplasts and zoospores with RNP complexes

**Fig. 1:**
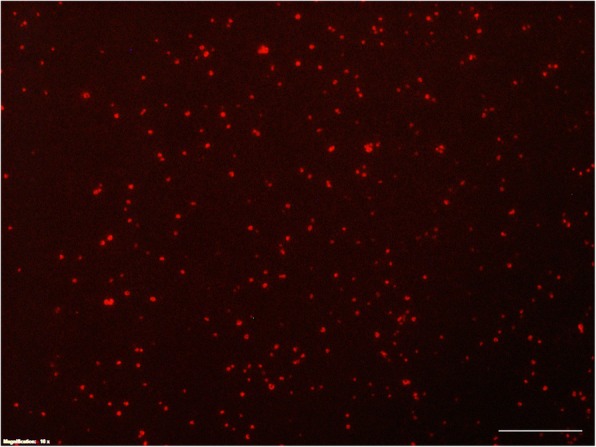
Fluorescence microscope image of the protoplasts of *Aphanomyces invadans*. Protoplasts were transfected with RNPs carrying ATTO^TM^ 550. Successful transfection shows clear RNP-complex signals within the protoplasts after overnight incubation. *Scale-bar*: 100 μm

**Fig. 2:**
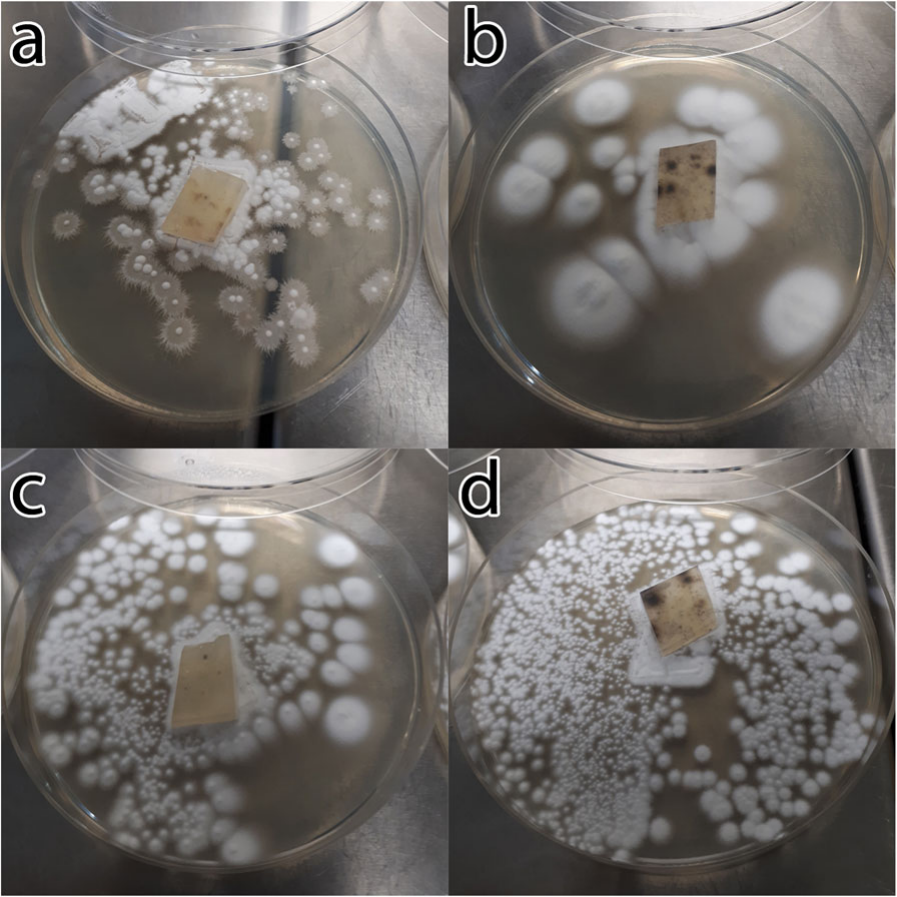
Growth of RNP-transfected *Aphanomyces invadans* protoplasts after being transfected with RNP-1 (**a**), RNP-2 (**b**) and RNP-3 (**c**). **d** Growth of non-treated control protoplasts

After the formation of RNP complexes, they were used separately to transfect the isolated *A. invadans* protoplasts. Examination of the successful transfection by fluorescence microscope revealed clear signals within the protoplasts (Fig. 1) and no signal in non-treated control samples. After regeneration of the RNP-treated protoplasts in GPY medium for 3–4 days, the protoplasts treated with the RNP-2 complex demonstrated a different growth pattern than those treated with RNP-1, RNP-3 and the non-treated control protoplasts (Fig. 2). Correspondingly, the RNP-treated zoospores demonstrated slow growth behavior in GYP medium compared with the non-treated controls.

### Detection of targeted mutation

**Fig. 3:**
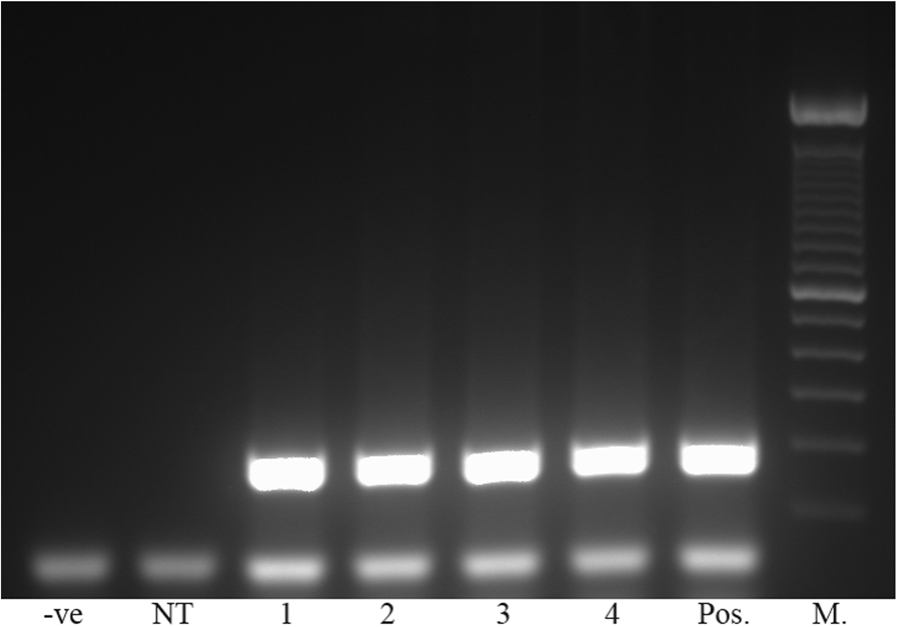
PCR result (214 bp) of RNP-2-treated and non-treated protoplasts and zoospores using primer set 2. Lane **-**ve: negative extraction; Lane NT: non-template; Lane 1: protoplasts transfected with RNP-2; Lane 2: non-treated protoplasts; Lane 3: zoospores transfected with RNP-2. Lane 4: non-treated zoospores. Lane Pos.: positive control. Lane M: marker (100 bp)

**Fig. 4:**
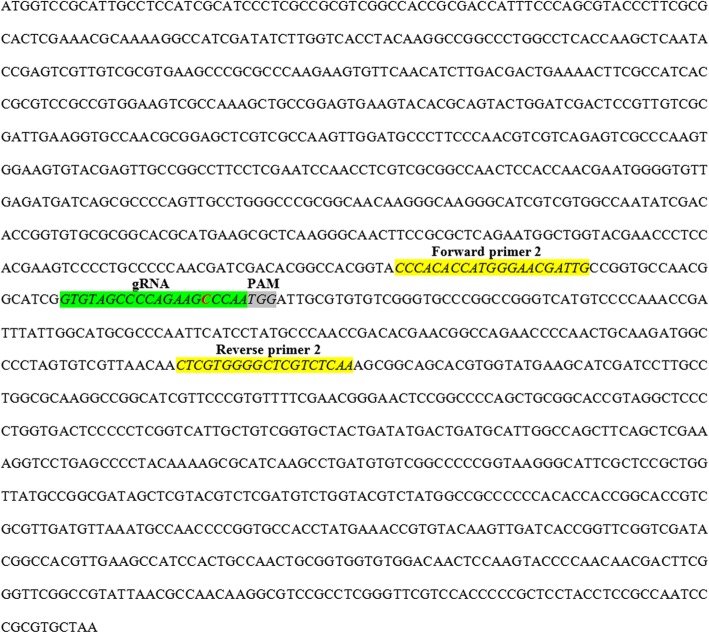
Coding sequence (mRNA sequence) of *Aphanomyces invadans*’ hypothetical protein (GenBank: XM_008877667) contains 1388 nucleotides. The designed gRNA2 site is highlighted in green and the PAM site in grey. Forward and reverse primer 2 (product size 214 bp) sites are highlighted in yellow. The point mutation (deletion) located at the 5th nt from the PAM site is shown in red within the gRNA2 sequence

The expected *A. invadans* DNA fragments were successfully amplified from RNP-treated and non-treated protoplasts and zoospores (Fig. 3). Sequencing results revealed a mutation located at the 5th nt from the PAM site in sequences of the targeted fragments amplified from protoplasts and zoospores treated with RNP-2 complex (Fig. 4). Comparatively, no mutation was detected in the sequences of protoplasts and zoospores treated either with RNP-1 or RNP-3.

### *In vivo* experiments

**Fig. 5:**
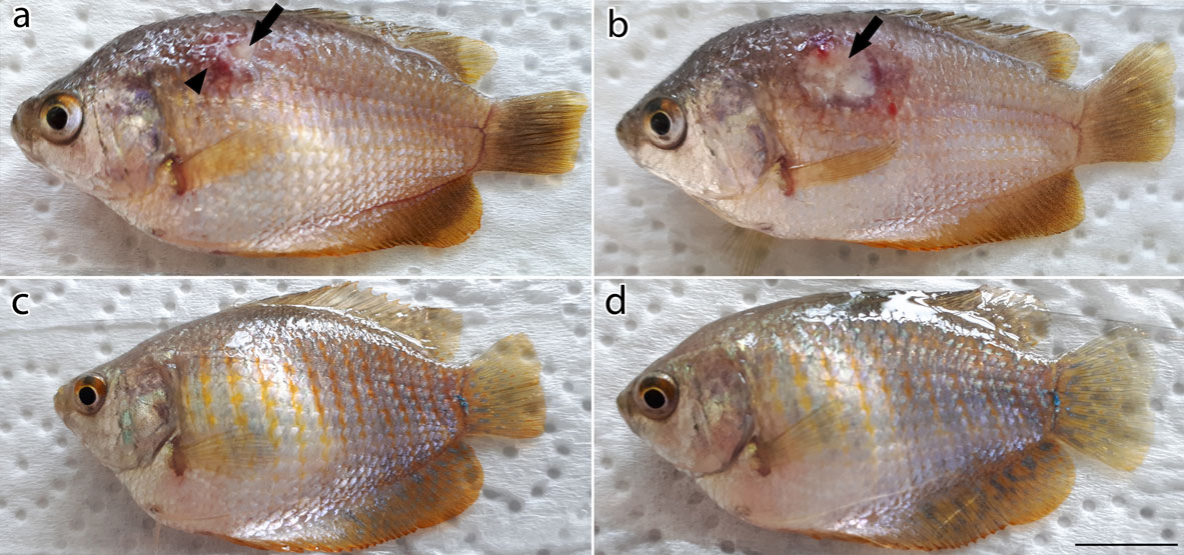
Dwarf gourami showing necrotic ulceration after infection with the zoospores of *A. invadans*. Zoospore suspension (0.1 ml) coniatining 10,000 zoospores/ml of *A. invadans* was injected intramuscularly. **a** Positive control fish showing the reddening of the infected area (arrowhead) and the beginning of ulcer formation (arrow) at the site of infection at 3 dpi. **b** Positive control fish showing clear oomycete growth and necrotic ulceration (arrow) at the site of infection at 7 dpi. **c** Fish injected with RNP-2-treated *A. invadans* zoospores showing no clinical signs at 7 dpi. **d** Negative control fish at 7 dpi. *Scale-bar*: 1 cm

At 3 days post-infection (dpi), fish within the positive control group inoculated with non-treated zoospores demonstrated swelling, reddening of skin and loss of scales; red ulceration underlying musculature was observed at 5 dpi. The ulceration further developed into deep ulceration with cotton-like threads at 7 dpi (Fig. 5a, b). Swimming behavior of the fish was affected and the fish showed weakness at 10 dpi. The presence of *A. invadans* DNA in the sampled fish tissues was confirmed by PCR and sequencing. Fish inoculated with the RNP-2-treated zoospores (Fig. 5c) and fish within the negative control group inoculated with APW (Fig. 5d) did not show any clinical signs during the 30 days of the experiment. PCR did not amplify the *A. invadans* DNA from the fish samples taken from these two groups. Negative control fish did not show any illness throughout the experiment.

### Histological investigation

**Fig. 6:**
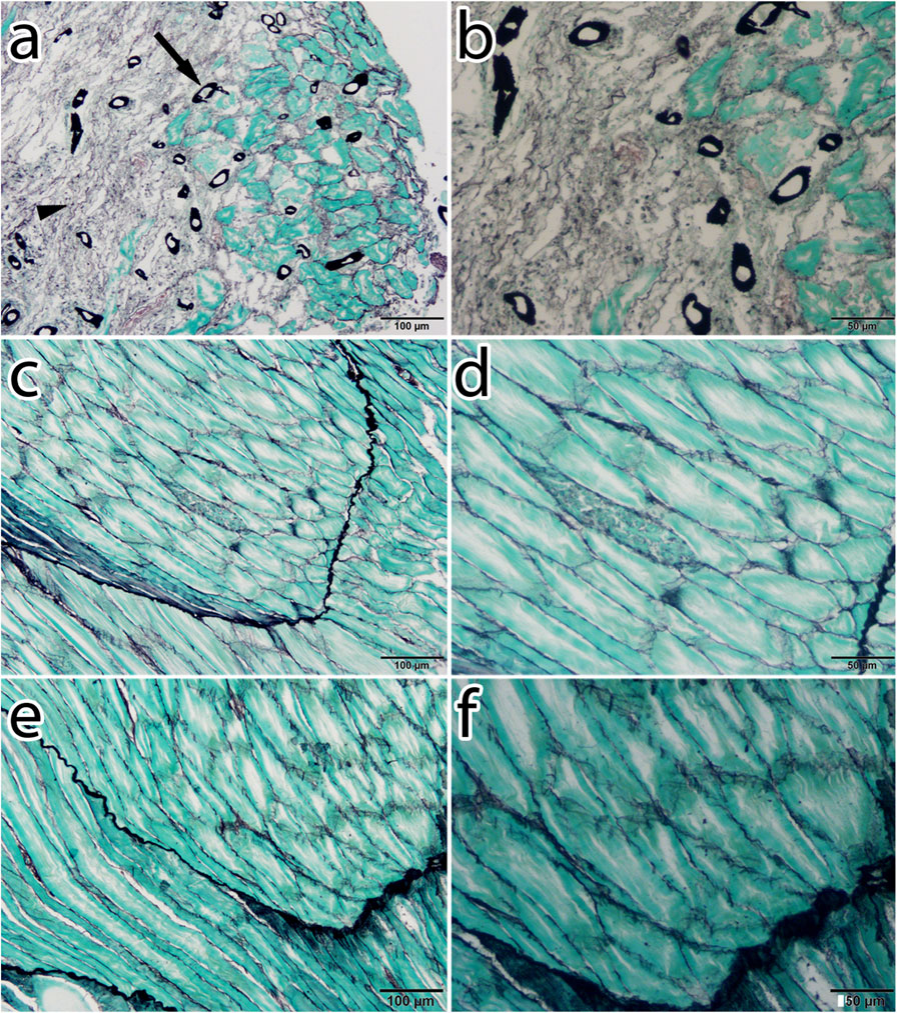
Gomori’s methenamine silver-stained muscle sections of dwarf gourami. **a** Muscle section of positive control fish showing presence of *A. invadans* as a black hollow structure (arrow) and muscle necrosis (arrowhead) in the gray area. **b** Higher magnification of **a**. **c** Muscle section of the fish inoculated with RNP-2-treated zoospores showing intact muscle with no oomycete hyphae. **d** Higher magnification of **c**. **e** Muscle section of negative control fish showing intact muscle with no oomycete hyphae. **f** Higher magnification of **e**. *Scale-bars*: **a**, **c**, **e**, 100 μm; **b**, **d**, **f**, 50 μm

**Fig. 7:**
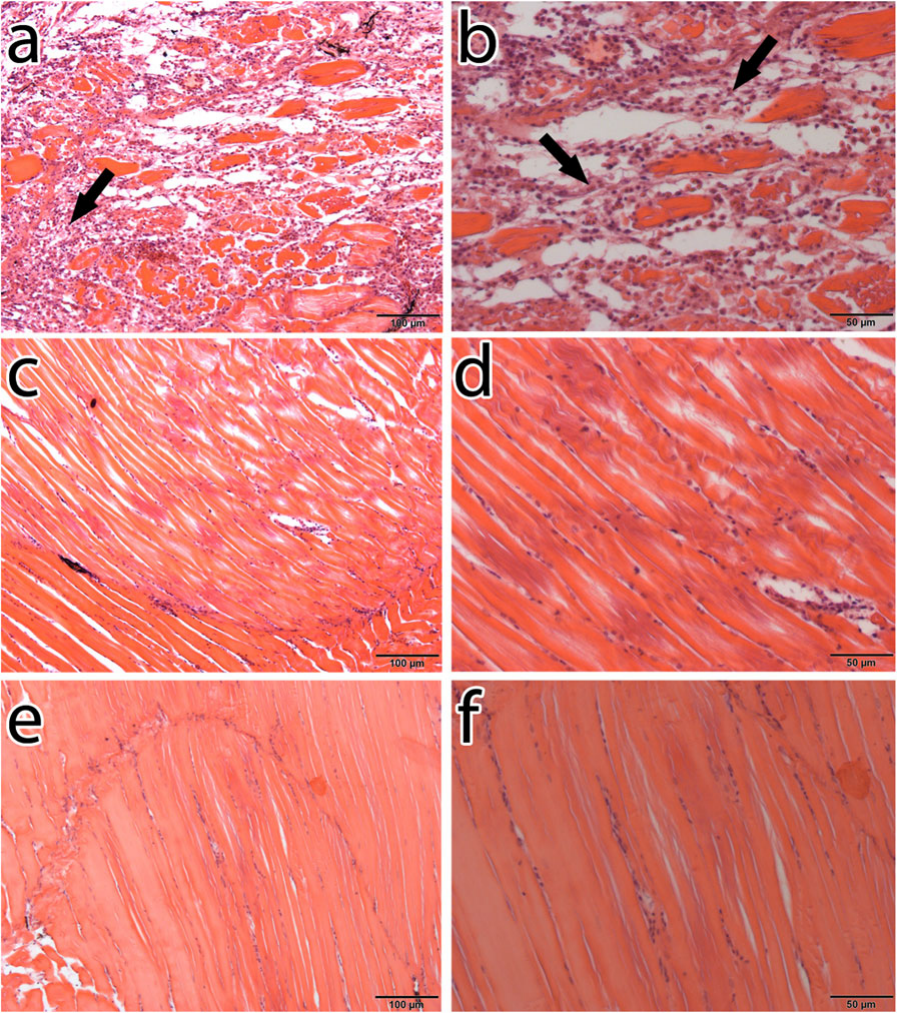
Hematoxylin and eosin-stained muscle sections of dwarf gourami. **a** Muscle section of positive control fish showing muscle necrosis (arrow). **b** Muscle section of positive control fish showing high infiltration of macrophages and other inflammatory cells (arrows). **c** Muscle section of the fish inoculated with RNP-2-treated zoospores showing no necrosis or infiltration of macrophages. **d** Higher magnification of **c**. **e** Muscle section of negative control fish showing no necrosis or infiltration of macrophages. **f** Higher magnification of **e**. *Scale-bars*: **a**, **c**, **e**, 100 μm; **b**, **d**, **f**, 50 μm

GMS-stained muscle sections of the fish from positive control group sampled at 7 dpi showed the presence of oomycete hyphae embedded into the fish musculature, with muscle tissues appearing necrotic around the hyphae in positive control group sampled at 7 dpi (Fig. 6a, b). No such fungal structure was found in the RNP-2-treated (Fig. 6c, d) or negative control groups (Fig. 6e, f). Sections of the fish muscles stained with H&E showed infiltration of macrophages and other inflammatory cells at the site of infection in positive control group (Fig. 7a, b). No such infiltration or necrosis of muscle tissues was found in the RNP-2-treated (Fig. 7c, d) or negative control groups (Fig. 7e, f).

## Discussion

Production of extracellular proteins from *A. invadans* has been reported by Majeed et al. [20]. These proteins mainly consist of proteases that are believed to be the major virulence factors that contribute to EUS infection. One of the identified proteins contains the peptidase_S8 domain which is a subtilisin or subtilase family, the second-largest family of serine proteases. Serine proteases are present in every life form including viruses and play major roles in protein metabolism, digestion, development, host invasion and immune evasion [54]. These proteases have been reported to be the virulence factors in different pathogenic species including fungi [55–57], parasites [58, 59] and bacteria [60–62]. Accordingly, in this study, this gene was selected for gene editing using CRISPR/Cas9 system in a trial to investigate its role in EUS. Currently, ZFNs and TALENs are used for genome editing experiments but the focus has been shifted to CRISPR/Cas system for such purposes. In the present study, the phenotype of germinating *A. invadans* protoplasts treated with three different RNP complexes demonstrated a different growth patterns. The PEG-mediated RNP delivery system is the most widely used system for delivering the gRNA and Cas9 into the protoplasts of oomycete and fungi. Based on fluorescence microscopy, the transfection resulted in bright and strong fluorescence signal which confirmed the efficiency of the transfection (Fig. 1). Nevertheless, obtained populations are expected to carry a mixture of mutated and non-mutated protease genes.

Sequence analysis of DNA of these protoplasts revealed the presence of point mutation in amplified fragments of the target gene caused by only one out of the selected three gRNAs. The same mutation was also detected in the zoospores treated with the same RNP complex (Fig. 4). These results confirm that only RNP-2 caused the mutation, while the RNP-1 and RNP-3 failed to perform similarly. The failure of the RNP-1 and RNP-3 to cause mutation may be attributed to the self-complementarity which likely hindered their binding to the target DNA sequence and affected their functionality [47]. The different growth pattern was only observed in the protoplasts that were treated with RNP-2 while the control and RNP-1- and RNP-3-treated protoplasts demonstrated a conventional growth pattern (Fig. 2). Furthermore, the observed slow growth pattern of the treated zoospores was likely because of the point mutation in the target gene. This indicates that the target gene may also play an important role in the growth of the *A. invadans*. This is supported by the fact that the target gene is a serine protease and serine proteases play major roles in regulation of development and host invasion [54]. Thus, any effective mutation in these genes will likely affect other cellular functions as well.

It seems that the target gene likely plays an important role in the virulence of the *A. invadans*. Dwarf guorami that were inoculated with non-treated zoospores demonstrated characteristic EUS clinical signs such as reddening, swelling, deep ulcers in skin and muscles, and visible fungal growth at the site of infection (Fig. 5a, b). On the other hand, fish that were inoculated with the RNP-2-treated zoospores did not demonstrate any clinical signs during the 30 days of the *in vivo* experiment (Fig. 5c). Mutation was confirmed *via* sequencing and all sequenced samples have this mutation. It was, however, confirmed by the *in vivo* experiments when the edited zoospores did not produce the EUS clinical signs. This shows that the zoospores carry the mutation.

GMS staining is the standard approach for the visualization of fungal agents in granulomatous inflammation [63, 64]. It stains the walls of the fungus and gives them dark/black coloration [53, 63]. GMS-stained histopathological sections of muscle tissues of the positive control fish at 7 dpi clearly showed the stained oomycete hyphae (Fig. 6a, b). Necrosis of muscle tissues were observed around the area of the hyphae, likely because of the intense inflammatory response [65]. Accumulation of macrophages and other inflammatory cells at the site of infection were also observed in H&E stained sections (Fig. 7a, b). Presence of *A. invadans* hyphae in fish musculature during EUS infection has been reported [14, 65] and our findings are in accordance with these results. Muscle sections of RNP-2-treated and negative control fish did not show any hyphae in GMS staining and no infiltration of inflammatory cells was seen in H&E stained sections of these fish at 7 dpi.

During CRISPR/Cas9-mediated genome editing, the suppression of the target gene by gRNA-complex appearently affects oomycete development and virulence, resulting in the cessation of *A. invadans* pathogenesis. The results obtained from this study hint on the fact that the serine proteases are definitely required by the oomycete in order to invade the fish and penetrate into the muscles. Protease production was likely affected in RNP-2-treated *A. invadans*, which might have hindered the production of other essential proteins as well. Thus, the oomycete was left unable to invade the fish musculature.

## Conclusions

In this study, despite lacking prerequisite information on molecular mechanisms of pathogenicity and interactions of *A. invadans* with the fish host, the CRISPR/Cas9 system was successfully applied to edit the serine protease gene of *A. invadan*s. Our study establishes that CRISPR/Cas9 is a promising tool and offers a great opportunity to plan more advanced genomic studies on oomycetes to understand the role of different secreted proteases, their virulence mechanisms, and their interactions with their hosts. In addition, our study provides a basis for further investigations of molecular mechanisms previously suggested to be involved in EUS and may help the development of novel drugs and control methods to combat their deleterious effects in fish.

## Abbreviations

APW: Autoclaved pond water
Cas9: CRISPR-associated protein 9
CRISPR: Clustered regularly interspaced short palindromic repeats
crRNA: CRISPR RNA
dpi: Days post-infection
EuPaGDT: Eukaryotic Pathogen CRISPR Guide RNA Design Tool
EUS: Epizootic ulcerative syndrome
GMS: Grocott’s methenamine silver nitrate stain
GP: Glucose peptone
GPY: Glucose peptone yeast
gRNA: Guide RNA
H&E: Hematoxylin and eosin
MES: 2-(N-morpholino)ethanesulfonic acid
PEG: Polyethylene glycol
RNP: Ribo-nucleo-protein
RT: Room temperature
TALEN: Transcription activator-like effector nuclease(s)
ZFN: Zinc finger nuclease(s)
